# An Application of Analytic Hierarchy Process and Entropy Weight Method in Food Cold Chain Risk Evaluation Model

**DOI:** 10.3389/fpsyg.2022.825696

**Published:** 2022-04-19

**Authors:** Yuyan Shen, Kaicheng Liao

**Affiliations:** ^1^Hangzhou Normal University, Hangzhou, China; ^2^School of Economics and Management, Tongji University, Shanghai, China

**Keywords:** analytic hierarchy process (AHP), entropy weight method, cold chain, risk assessment, evaluation model

## Abstract

The food cold chain is a special type of cold chain that refers to a system in which refrigerated and frozen food is always kept in the specified low-temperature environment in all links from production, storage, transportation, sales, distribution to consumption, so as to ensure food quality and to prevent food deterioration caused by temperature fluctuation. In recent years, the coronavirus disease 2019 (COVID-19) has brought a great impact on people’s life and the social economy and also threatened the large-scale food cold chain. Through the effective identification and evaluation of high-risk factors in the food cold chain, this article has found the major risks that have a great impact on the entire food cold chain and proposes the specific measures of risk management and control to solve the problems of food cold chain and reduce risks quickly and efficiently to ensure the stability and safety of food cold chain and avoid the serious food safety accidents. The contribution of this article is reflected in three aspects, namely, (1) applies the expert system based on professional knowledge and rich experience and constructs a classification and identification system structure of food cold chain risk indexes, which lay a foundation for further identifying and evaluating the major risks of the food cold chain; (2) designs a comprehensive index weighting method combining the AHP method and entropy weight method to quantitatively evaluate the major risks. This comprehensive method combines a hierarchical structure system, evaluation algorithm, subjective factor correction algorithm, and so on. The evaluation results are more accurate, have a high matching degree with reality, and have good theoretical and practical significance; (3) analyzes and explains the major risks of the food cold chain in the non-epidemic situations and COVID-19 situations. Proposals and measures for risk management and control are put forward, which have wide practical significance.

## Introduction

The food cold chain plays a vital role in satisfying the growing demand for perishable foods (Ovca and Jevsnik, 2009). An improper food cold chain management increases the possible risk of potential microbial hazards, which may lead to foodborne illnesses (Ucar and Ozcelik, 2013). In this article, the term food cold chain is a special type of cold chain that refers to a system in which refrigerated and frozen food is always kept in the specified low-temperature environment in all links from production, storage, transportation, sales, distribution to consumption, so as to ensure food quality and to prevent food deterioration caused by temperature fluctuation. In the food cold chain, refrigerated products include fresh dairy products, cooked products, fruits, vegetables, eggs, meat products, and aquatic products, and frozen products include all kinds of meat, seafood, and ice cream. Food cold chain products are restricted to food and do not include non-food products such as pharmaceuticals, vaccines, and chemicals. In recent years, the coronavirus disease 2019 (COVID-19) has brought a great impact on people’s life and the social economy and also threatened the large-scale food cold chain. Since the outbreak of the global COVID-19, the food cold chain logistics has faced huge risks, and the security incidents have emerged one after another. Frozen chicken wings, frozen shrimp, cherries, and other foods imported into China from overseas have been detected to carry the COVID-19, which has aroused great concern about the safety of cold chain logistics (Yan, 2021). The large-scale cold chain serves thousands of households, whether it is a modern city or a remote rural area. Moreover, the food cold chain has become an indispensable support system for people’s quality of life (Kuo and Chen, 2010). Any problems occurring in the cold chain can cause deterioration in products, causing poisoning, death, or various diseases (Dagsuyu et al., 2021). Ensuring the safe, reliable, stable, and efficient operation of the food cold chain is important in the context of the global epidemic particularly. The cold chain system has applied a lot of new technologies (Tsang et al., 2018; Villalobos et al., 2019), with a large geographical span, high-temperature control requirements, many cooperating entities, and complex internal structures. Many links generate risks and many risk incentives; it is difficult for the risk management and control with the wide-ranging risks.

At present, in the context of the global epidemic, the probability of contamination of products in the food cold chain has increased significantly. According to public media reports, the virus survives for a long time in a low-temperature environment, which intensifies the potential risk of the food cold chain (Sobolik et al., 2021). If the products are polluted and there is a lack of timely and efficient control, it will bring major safety accidents to the whole cold chain. How to identify and evaluate high-risk factors in the food cold chain, find the major risks that have the greatest impact on the entire chain, and prevent the major food safety accidents with wide-ranging impacts effectively? (Guo et al., 2018). It is imperative to manage and control the risks and solve the cold chain risks quickly and efficiently. The construction of an evaluation model for the importance of risk indexes in the food cold chain is of great significance for controlling all aspects of the cold chain and ensuring people’s livelihood effectively.

Identifying and assessing high-risk factors in the cold chain is an effective method to prevent the major food safety accidents with widespread coverage. The research on food cold chain risk assessment methods and models focuses on two aspects, namely, the qualitative identification and analysis of cold chain logistics risks, and the quantitative establishment of cold chain risk assessment models. Raut et al. (2019) have used the multicriteria comprehensive decision-making method (MCDM) to solve the problems related to the evaluation of third-party cold chain logistics (3PL) suppliers. Nakandala et al. (2017) have used a hybrid model combined with risk filtering, ranking and management (RFRM) fuzzy logic (FL) to evaluate the risk of the fresh food supply chain. Srivastava et al. (2015) have analyzed the relationship between the risk and performance indexes in the fresh food retail supply chain based on the explanatory structural model Interpretative Structural Modeling Method (ISM) and explained the spread of risk in fresh food retail. Although there are many risk assessment methods applied to the risk assessment of the cold chain, most of these research methods lack objective evaluation and do not consider the impact of exogenous risks, so it is difficult to avoid the subjective biases in the decision-making process and final results (Zhang et al., 2017); there is still room for optimization in terms of index selection and weighting methods. COVID-19 is an environmental factor with high risk in recent years, that is devastating, spreading widely and adapting to the ultra-low-temperature environment, which brings huge risks to the food cold chain. The research gap shows that the cold chain itself is an extremely complex system, involving many risk factors (Peng et al., 2017). Therefore, it is necessary to use the comprehensive evaluation method to conduct a comprehensive risk factor analysis of the cold chain and find the systemic problems (Janjanam et al., 2021). There is an urgent need to improve and optimize the index selection and weighting methods. The purpose of this article was to find an effective risk assessment method and prevent systemic risks in the cold chain.

Overall, the research value of this article is as follows. (1) Risk identification and assessment of the food cold chain is not only a subject of great practical significance but also a difficult problem. The food cold chain is a huge comprehensive system with a complex structure, many operation links, many related factors, strong dependence on professional technology, large fluctuations in product supply and demand, a large number of consumers, fierce competition, and difficult risk management (Chen and Wang, 2020). Based on the theme of food cold chain risk identification and evaluation, this article constructs a multilevel risk hierarchy, establishes a comprehensive evaluation model, and puts forward risk management and control suggestions. It plays an active role in the research, maintenance, operation, and popularization of the food cold chain. (2) This study creatively constructs a comprehensive quantitative evaluation model to effectively identify and evaluate the major risks of the food cold chain, by using the expert system based on rich knowledge and professional experiences and combining the AHP method and entropy weight method, and takes China’s food cold chain as the research object and gets some important results. The analytic hierarchy process (AHP) is used to evaluate expert systems quantitatively through hierarchical structure and professional algorithms, and the entropy weight method is introduced to reduce the negative effect of individual subjective evaluation bias on the accuracy of comprehensive evaluation (Al-Harbi, 2001; Dyer and Forman, 1992). This model combines the advantages of subjective and objective evaluation to make the results more accurate, reasonable, and effective. (3) Based on the quantitative evaluation results, this study analyzes and explains the major risks of China’s food cold chain in the case of the non-epidemic situation, and gives proposals and measures to prevent and control risks, which has extensive reference value and practical significance. At the same time, this article also analyzes and explains the mechanism of the special risks of the food cold chain under the situation of COVID-19, which contributes to preventing and controlling the risks caused by COVID-19.

In what follows, we have first conducted a literature review of “Risk Causing Factors of Cold Chain” and “Risk Assessment Model of Cold Chain” given in the “Literature Review” section. Subsequently, we have introduced research materials and methods in the “Index Selection and Evaluation Method” section. Then, we have chosen China’s food cold chain as a case study to analyze in the “Empirical Results” section. Finally, the discussion and prospects are presented in the “Discussion” section. Based on the risk index value of China’s food cold chain, this article analyzes 18 specific indexes of five parts, ranks the risks of each link of the food cold chain, and finally puts forward corresponding countermeasures and proposals.

## Literature Review

It is very important to coordinate and manage all node enterprises in the cold chain. We should have a strong risk awareness and strive to identify, evaluate, and control risks, to improve the robustness of the whole food cold chain, to ensure food safety, and to improve the performance of the food cold chain. The food cold chain is a systematic project, and its circulation involves many enterprises (Hsiao et al., 2018). Although the dependence among members of the food cold chain brings many benefits to the organization, it increases the uncertainty and risk in cold chain activities (Guo et al., 2018). The food cold chain requires that the whole process should be controlled under low-temperature conditions (Lau et al., 2021). Scholars’ studies have shown that the whole-process temperature control and delivery timeliness are the most critical factors affecting food cold chain risks. If the risks are not identified and controlled, food safety accidents will be directly caused. Earlier studies have pointed out that efficient temperature monitoring and effective temperature data management are important prerequisites for providing high-quality and safe products and avoiding economic losses throughout the cold chain (Wu and Hsiao, 2021). The quality and safe storage of food in the cold chain are closely related to factors such as temperature and humidity in the supply chain environment (Taoukis et al., 2016). It also includes specific transaction and process-related data such as pickup rates, transfer times from site to chiller, transportation costs, and vehicle routing issues with time windows (Chaudhuri et al., 2018).

Further research by scholars has shown that for fresh food, ensuring the stability of the supply chain is extremely important, especially the control of time delay and temperature variation range (Mercier et al., 2017). Risk management of the cold chain must start from the production process, and for the perishable food, the key dimensions of cold chain management such as information technology, equipment, infrastructure, and regulation must be interconnected (Bremer, 2018). The evaluation of supply chain risk indexes should fully consider the risks existing in each link of the supply chain (Zhang and Liao, 2019), at the same time, enhance the connection of each link and the ability of logistics operation to reduce the possibility of risks (Xu, 2020).

The research on cold chain risk assessment methods and models focuses on the establishment of cold chain risk assessment models. A hybrid model of Fuzzy AHP and Fuzzy TOPSIS is proposed for the selection of an appropriate 3PL to outsource logistics activities of perishable products (Singh et al., 2018) and to identify and assess supply chain risk across multiple product categories using cognitive maps and AHP methodology (Mital et al., 2018). Scholars have used AHP, fuzzy comprehensive evaluation, and others to carry out identification and evaluation research (Aqlan and Lam, 2015), but most of these research methods require the participation of subjective opinions and lack objective evaluation (Yan et al., 2014; Butdee and Phuangsalee, 2019; Ganguly and Kumar, 2019; Junaid et al., 2020), and it is still difficult to avoid subjective bias in the decision-making process and final results (Zhang et al., 2017). Although there are lots of risk assessment models applied to different risk assessment projects (e.g. Joshi et al., 2009; Shambayati et al., 2020), the AHP especially has a wide application in many situations. However, the application of AHP in the risk assessment of the cold chain is not sufficient. The entropy weight method is an objective weighting method for the index weight, which avoids the defect of overemphasizing human subjective judgment in the subjective weighting method to a certain extent (Li et al., 2014).

Therefore, in the selection of food cold chain risk indexes, the article has introduced the environmental risk factors of the COVID-19 to modify the food cold chain risk indexes, and experts and senior practitioners in this field were invited to conduct the systematic evaluation by scoring, to further find the systemic risk problems of the cold chain. Combining the subjective experience value of the AHP (Mital et al., 2018) and the objective information entropy value of the entropy method, the index weighting method can calculate the risk index score more reasonably, so that the risk assessment model of the food cold chain can be more consistent with the reality and effective use of information. According to the results of risk assessment, some effective propositions are shown to reduce major risks in the food cold chain under normal background and COVID-19 background. The article has found the major risks that have the greatest impact on the entire cold chain by evaluating the risk factors of the food cold chain effectively. It can be based on evidence, focus on solving the main contradictions, and solve the supply chain risk problems more quickly and effectively. At the same time, through the research on the relationship among various risk factors, the synergistic impact of key risk factors and other factors is further explored, the impact of risk factors on the food cold chain is minimized, and specific risk control measures are proposed to improve the stability of the entire food cold chain.

## Index Selection and Evaluation Method

### Index Selection Basis for Food Cold Chain Risk System

**FIGURE 1:**
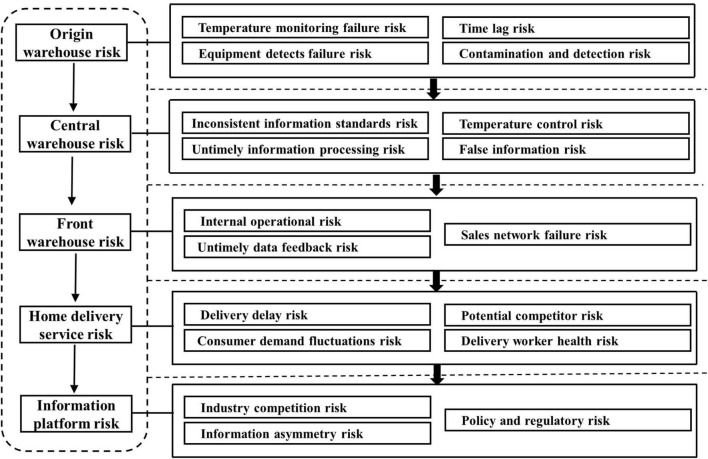
Risk factors of the food cold chain.

Food cold chain refers to the system that refrigerated and frozen food is always in the specified low-temperature environment in the whole process of production, storage, transportation, reprocessing, and sales, to ensure food quality and safety, reduce consumption, and prevent food contamination and deterioration. Maintaining a low-temperature environment is the core requirement (Fu et al., 2019). Following the principles of comprehensive, objective, dynamic, qualitative, and quantitative combination with recommendations from industry experts, in line with national cold supply chain industry standards, the major risk indexes of the food cold chain can be determined. This article identifies and classifies the risks of food cold chain in non-epidemic environment, mainly from five aspects, namely, origin warehouse risk, regional center warehouse risk, front warehouse risk, home delivery service risk, and information platform risk. Finally, the special risks of COVID-19 are discussed, as shown in Figure 1.

➀ Origin warehouse risk. When purchasing and storing products at the place of origin, strict source quality control shall be carried out on production environment, taste, appearance, and quarantine. The application of Internet of things (IoT) technology may be used to collect and process the information of source products and adopt an identification system and traceability system to strictly control the quality of products in an all-round way. The major risks include temperature monitoring failure risk, detection equipment failure risk, time lag risk, and contamination and detection risk.

➁ Regional central warehouse risk. The regional central warehouse is an important core of the food cold chain, which has a large storage capacity, long storage time of goods, high-temperature control requirements, highly centralized information including scheduling and distribution information, and a wide range of influence. The major risks include temperature control risk, false information risk, inconsistent information standards risk, and untimely information processing risk.

➂ Forward position risk. The front warehouse is close to the consumer group, and its maintenance cost is relatively high. The type and quantity of goods will be adjusted in time according to the actual consumption demand and predicted consumption demand. In contrast, it maintains information sharing within the regional central warehouse, improves the operation efficiency of the front warehouse, and reduces inventory loss. The major risks include internal operation management risk, sales network failure risk, and untimely data feedback risk.

➃ Home delivery services. Excellent home delivery service can maximize users’ consumption experience of “rapid delivery” and stimulate users’ consumption demand, to greatly improve the brand awareness of enterprises, expand market share, and improve economic benefits. The link of the home delivery can realize direct communication and contact with consumers, understand the real needs of consumers, and promote enterprises to optimize operation strategies in time, reduce losses, and improve profits. It is precise because of the importance of home delivery that there is a fierce business competition, which affects the future and destiny of cold chain enterprises. The risks of home delivery service include delivery delay risk, consumer demand fluctuation risk, potential competitors risk, and delivery worker health risk.

➄ Industry information platform risks. The industry information platform is an information processing center, which can analyze and process product information, vehicle information, refrigeration parameter, traffic operation data, and transaction data. In the case of COVID-19, an excellent industry information platform helps prevent and control risks. The main service objects of the platform are government departments, refrigerated transportation enterprises, warehousing and distribution enterprises, and so on. The industry information platform risks include information asymmetry risk, industry competition risk, and policy and regulatory risk.

### Selection of Specific Indexes

Referring to China’s national standards for the cold chain industry and based on the basic structure of the food cold chain, the main indexes of it are identified according to the principles of comprehensiveness, objectivity, dynamics, qualitative and quantitative combination, and the suggestions of industry experts. Five primary indexes and eighteen secondary indexes were identified, as shown in Table 1.

**TABLE 1:** Description of risk indexes of food cold chain.

No.	Rule layer	Factor layer	Sign	Index description
1	Origin warehouse (X_1_)	Temperature monitoring failure risk	X_11_	Lead to risk of spoilage
2		Equipment detect failure risk	X_12_	Lead to quality risks
3		Time lag risk	X_13_	Lead to the decreased freshness of fresh products
4		Contamination and detection risk	X_14_	The quality of fresh products is out of control
5	Regional central warehouse (X2)	Inconsistent information standards risk	X_21_	Lead to risk of loss of product quality control
6		Untimely information processing risk	X_22_	Lead to risk that the product backlog
7		Temperature control risk	X_23_	Lead to risk of loss of product quality control
8		False information risk	X_24_	Lead to risk that the product backlog
9	Front warehouse (X3)	Internal operational risk	X_31_	Lead to poor logistics and reduce product quality
10		Untimely data feedback risk	X_32_	Lead to risk that the product backlog
11		Sales network failure risk	X_33_	Lead to risk that the product backlog
12	Home delivery service (X4)	Delivery delay risk	X_41_	Lead to risk of poor logistics
13		Consumer demand fluctuations risk	X_42_	Lead to risk that the product backlog
14		Potential competitor risk	X_43_	Lead to risks such as product backlog and poor logistics
15		Delivery worker health risk	X_44_	Lead to risk of product service not being in place
16	Industry information platform (X5)	Industry competition risk	X_51_	Lead to risks such as product retention and poor logistics
17		Information asymmetry risk	X_52_	Lead to risk of unsalable products
18		Policy and regulatory risk	X_53_	Lead to risks such as high logistics costs

Using the Delphi method, through the scoring and evaluation of 10 experts in the field of food cold chains, the risk assessment index system is constructed. This article also considers the impact of environmental factors (Saif and Elhedhli, 2016) of COVID-19 on risk indexes and makes further discussions at the end.

### Evolution Methods

**FIGURE 2:**
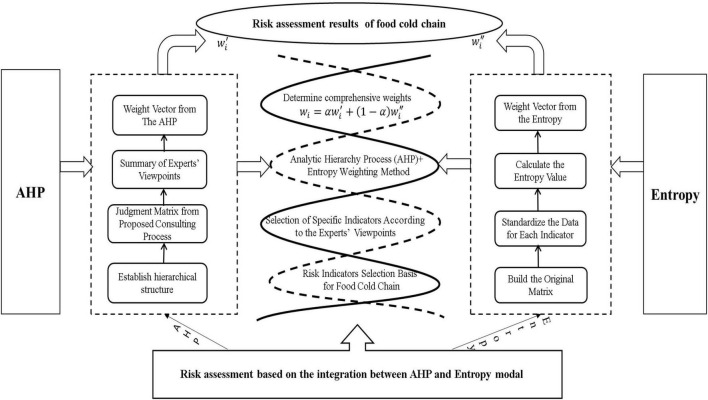
Flow pipe of the risk assessment using AHP-entropy methods.

The analytic hierarchy process and Delphi belong to the subjective weighting method; the entropy method and principal component analysis belong to objective weighting method. In this article, the AHP and entropy weight method were used to determine the weight of each index, and the weighted average was used to calculate the comprehensive weight of the two methods (Mital et al., 2018; Ganguly and Kumar, 2019), to avoid the large comprehensive weight result caused by the difference between subjective factors or objective factors. The influence degree of the two methods was analyzed according to the actual situation; the weight coefficients of the two methods were determined, and finally the comprehensive weight was calculated, as shown in Figure 2.

#### Analytic Hierarchy Process

The analytic hierarchy process is a multicriteria decision-making method combining qualitative and quantitative analysis proposed by American operations’ research scientist Saaty (1977). The risk of the food cold chain is complex and diverse, which requires a combination of qualitative and quantitative methods to make a reasonable assessment (e.g., ranking, screening, and weight distribution). AHP is a useful way to analyze complex problems by dividing them into three layers, namely, object layer, rule layer, and factor layer (Saaty, 2000, 2003). When we assess the risks of some events, the risk is generally regarded as the object layer. Therefore, the first layer is the object layer, which indicates the risks of the food cold chain in this article. The second layer is determined by China’s national standards and the basic structure of cold chain industry, combined with the opinions of industry experts, which states that risk assessment of the food cold chain should be assessed from the following aspects: origin warehouse, regional center warehouse, front warehouse, home delivery service, and information platform. The third layer is a further subdivision of the second layer. According to the experts’ responses, 18 major risks points in the factor layer were identified. Based on the information and analysis earlier, a hierarchical structure for risk assessments of the food cold chain was established.

After establishing the AHP model, the subordinate relationship between the upper and lower levels was determined, and then the risk judgment matrix of each level element was constructed according to the AHP model and expert system. The weight of risk indexes the influence degree of the risk in the food cold chain risk assessment system. In this article, through the scoring of experienced experts in the food cold chain, the risk judgment matrix of each level was obtained, the weight of each factor under this level was calculated, and finally the weight of each level to the overall goal was calculated.

**FIGURE 3:**
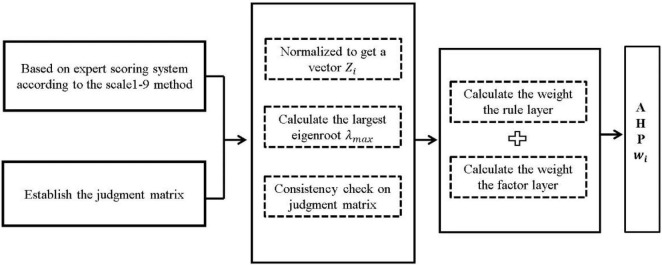
The calculation process of AHP method.

As shown in Figure 3, the calculation process of the AHP method is as follows:

(1) The AHP model is established to determine the relationship between the upper and lower layers, and the judgment matrix is constructed through the pairwise comparison between the factors of each layer. *X* = {*X*_1_,*X*_2_,*X*_3_}*X*_1_ = {*X*_11_,*X*_12_,*X*_13_,*X*_14_}*X*_2_ = {*X*_21_,*X*_22_,*X*_23_,*X*_24_}*X*_3_ = {*X*_31_,*X*_32_,*X*_33_}*X*_4_ = {*X*_41_,*X*_42_,*X*_43_,*X*_44_}*X*_5_ = {*X*_51_,*X*_52_,*X*_53_,*X*_54_}.

The judgment matrix is determined by the expert group in relevant fields according to the scale method of levels 1–9, as shown in Table 2.

**TABLE 2:** Scale 1–9 method of judgment matrix.

Scale	Meaning
1	Comparing two factors with equal importance
3	Comparing the two factors, the former is slightly more important than the latter
5	Comparing the two factors, the former is more important than the latter
7	Comparing the two factors, the former is much more important than the latter
9	Comparing the two factors, the former is definitely more important than the latter

*2, 4, 6, and 8 are the scale values corresponding to the intermediate states between the above two judgments.*

X is the judgment matrix:


(1) $$X=x_{m\,m}\,\left[\begin{array}{cccc} x_{11} & x_{12} & \cdots & x_{1\,m}\\ x_{21} & x_{21} & \cdots & x_{2\,m}\\ \vdots & \vdots & \ddots & \vdots \\ x_{m\,1} & x_{m\,2} & \cdots & x_{m\,m} \end{array}\right]$$

(2) Normalized to get a vector *Z_i*:

(2) $$Z_{i}=\sqrt[{m}]{x_{i\,1}\,x_{i\,2}\,x_{i\,3}\,\ldots \,x_{i\,m}}$$

The weight coefficient of the *i*-th index*wi*:


(3) $$w_{i}=\frac{Z_{i}}{\sum_{i=1}^{m}Z_{i}}$$

(3) Calculate the largest Eigen root of the judgment matrix λ_*max*_:

(4) $$\lambda_{\max}=\sum_{i=1}^{m}\frac{{\left(X\,w\right)}_{i}}{n\,w_{i}}$$

(4) Consistency check on judgment matrix:

(5) $$C\,I=\frac{\lambda_{\max}-n}{n-1}$$

(6) $$C\,R=\frac{C\,I}{R\,I}$$

Among them, *CI* is the consistency index, and RI is the average random consistency index of the matrix. When *CI* = 0, the judgment matrix has completion consistency; the larger the *CI* value, the worse the degree of consistency. When *CR* ≤ 0.1, the positive and negative judgment matrix has an acceptable degree of consistency; otherwise, the judgment matrix needs to be readjusted. The *RI* values of the 1–9 order judgment matrix are shown in Table 3.

**TABLE 3:** Average random consistency index.

n	1	2	3	4	5	6	7	8	9
*RI*	0	0	0.58	0.90	1.12	1.24	1.32	1.41	1.45

#### Entropy Weight Method to Establish Risk Factor Evaluation System

The entropy weight method can judge the randomness and disorder degree of an event by calculating the entropy value, and it can also use the entropy value to judge the discrete degree of an index. The greater the discrete degree of the index, the greater the impact of the index on the comprehensive evaluation. The weight is determined by the calculation of entropy, that is, the weight of each index is determined according to the different degree of the risk factor value.

The calculation process of the entropy weight method is as follows:

(a). Build the original matrix. Assuming that there are *m* objects to be evaluated and *n* evaluation indexes, the original matrix *X* can be constructed:

(7) $$X=\left[\begin{array}{cccc} x_{11} & x_{12} & \cdots & x_{1\,m}\\ x_{21} & x_{22} & \cdots & x_{2\,m}\\ \vdots & \vdots & \ddots & \vdots \\ x_{n\,1} & x_{n\,2} & \cdots & x_{n\,m} \end{array}\right]$$

Among them,*X*_*ij*_ represents the *i*-th risk, and the actual value of the *j*-th index.

(b). Data standardization. Standardize the data for each index:

(8) $$Y_{i\,j}=\frac{x_{i\,j}-m\,i\,n\,\left(x_{i}\right)}{m\,a\,x\,\left(x_{i}\right)-m\,i\,n\,\left(x_{i}\right)}$$

*Y*_*ij*_ is normalized:


(9) $$p_{i\,j}=\frac{Y_{i\,j}}{\sum_{i=1}^{n}Y_{i\,j}}$$

(c). Calculate the entropy value *E_j* of the *j*-th index:

(10) $$E_{j}\;=\;-\frac{1}{ln(m)}\sum_{i=1}^{n}pij\;\;lnpij$$

If *p*_*ij*_ = 0, and then define *p*_*ij*_*lnp*_*ij*_ = 0

(d). Calculate the entropy weight *w_i* of the *i*-th index:

(11) $$w_{i}=\frac{1-E_{j}}{m-\sum_{i=1}^{m}E_{j}},\left(i=1,2,3,\cdots ,m\right)$$

#### Establish Comprehensive Weights

Analytic hierarchy process is subjective and arbitrary, but it is systematic and explanatory. The weight obtained by the entropy weight method sometimes may not match or even contradict the actual importance, but the weight is highly objective, and it is not easily affected by subjective factors adversely. Therefore, by the combination method to combine the subjective and objective weights effectively, the combination of subjective judgment and objective data is realized, so that the evaluation results are more realistic.

By the AHP entropy weight method, the comprehensive weight of the evaluation index is determined as:


(12) $$w_{i}=\alpha \,w_{i}^{\prime }+\left(1-\alpha \right)\,w\,{"}_{i}$$

In the formula, α is the ratio of AHP to the combined weight, $w_{i}^{\prime }$ is the weight of each item calculated by the AHP, (1−α) is the ratio of the entropy weight to the combined weight, and *w*"_*i*_ is the entropy weight calculation various weights.

## Empirical Results of Risk Assessment of Food Cold Chain in China

### Data Selection

Considering the diversity and complexity of food cold chain risk, this article makes a reasonable evaluation by using qualitative and quantitative methods. According to the assignment rules, this article invites experts and senior practitioners with rich professional knowledge or rich industry experience to give the values of various elements of the judgment matrix. Finally, the results of China’s food cold chain risk assessment are calculated by the AHP enterprise method.

Specifically, we invited 10 experienced experts in the cold chain industry, including 4 senior managers who have long worked in well-known cold chain enterprises in China. They have more than 15 years of working experience in the cold chain industry and have participated in the formulation of relevant standards in the cold chain industry; 4 scholars and professors, who have been engaged in research on supply chain management for a long time; and 2 senior employees in the cold chain industry are familiar with the business of all links of the cold chain. The expert group comprehensively analyzes and evaluates the risk factors of the food cold chain, compares the importance of food cold chain risk factors at each level, and scores them according to the nine point method (from 1 = lowest importance to 9 = highest importance).

According to the principles of comprehensive, objective, dynamic, qualitative and quantitative, this article refines the influencing factors of the five modules of food cold chain risk. The food cold chain risk index system is divided into three levels, namely, target level, criterion level, and index level. Food cold chain risk index system as the target layer; the criterion layer includes five first-class indexes, namely, origin warehouse risk, regional center warehouse risk, front warehouse risk, distribution to household risk, and information platform risk; the primary indexes are further divided into 18 secondary indexes such as temperature control risk, distribution delay risk and consumer demand fluctuation risk.

### Calculate Analytic Hierarchy Process Weights

Following the food cold chain risk index system as shown in Table 2, combined with the expert group, the importance of the index layer by layer from top to bottom is scored and the weight is calculated according to the calculation steps of AHP method. Next, the rule layer is taken as an example to illustrate the calculation process. First, the subjective weight of the index given by each expert is calculated according to formulas (1)–(6), and the consistency is checked. The calculation results are shown in Table 4.

**TABLE 4:** Subjective weight calculation of rule layer matrix.

Rule layer	Judgment matrix	Consistency test	Weight
Origin warehouse	$\text{X}\left[\begin{array}{l} 1\;\;\;1/5\;\;\;1\;\;\;1/7\\ 5\;\;\;\;\;1\;\;\;\;\;\;5\;\;\;\;\;3\;\;\\ 1\;\;\;1/5\;\;\;1\;\;\;\;\;1/3\\ 7\;\;1/3\;\;\;3\;\;\;\;\;1 \end{array}\right]$	λ_*max*_ = 4.1992; *CI* = 0.0664; *CR* = 0.0738 <0.1	0.1186 0.4678 0.1229 0.2908
Regional central warehouse	$\left[\begin{array}{l} 1\;\;\;1/2\;\;\;1/5\;\;\;1/3\\ 2\;\;\;\;\;1\;\;\;\;\;\;1/6\;\;\;\;\;1\;\;\\ 5\;\;\;\;\;6\;\;\;\;\;\;\;\;1\;\;\;\;\;\;\;\;4\\ 3\;\;\;\;\;1\;\;\;\;\;\;\;1/4\;\;\;\;\;1 \end{array}\right]$	λ_*max*_ = 4.1176; *CI* = 0.0392; *CR* = 0.0436 <0.1	0.0754 0.1273 0.6408 0.1565
Front warehouse	$\left[\begin{array}{l} 1\;\;\;\;1/2\;\;1/4\\ 2\;\;\;\;\;\;1\;\;\;\;\;\;1/3\\ 4\;\;\;\;\;\;3\;\;\;\;\;\;\;1 \end{array}\right]$	λ_*max*_ = 3.0931; *CI* = 0.0466; *CR* = 0.0517 <0.1	0.1708 0.2739 0.5553
Home delivery services	$\left[\begin{array}{l} 1\;\;\;\;1/8\;\;\;1/9\;\;\;1/5\\ 8\;\;\;\;\;\;1\;\;\;\;\;\;\;\;\;1\;\;\;\;\;\;\;\;\;2\\ 9\;\;\;\;\;\;1\;\;\;\;\;\;\;\;\;1\;\;\;\;\;\;\;\;2\\ 5\;\;\;\;\;1/2\;\;1/2\;\;\;\;\;1 \end{array}\right]\;\;\;$	λ_*max*_ = 4.0047; *CI* = 0.0016; *CR* = 0.0017 <0.1	0.0220 0.3555 0.4156 0.2069
Industry information platform	$\left[\begin{array}{l} 1\;\;\;\;1/6\;\;\;1/5\\ 6\;\;\;\;\;\;\;1\;\;\;\;\;\;\;\;\;2\;\\ 5\;\;\;\;\;1/2\;\;\;\;\;1\;\;\;\;\; \end{array}\right]\;\;\;$	λ_*max*_ = 3.1608; *CI* = 0.0804; *CR* = 0.0893 <0.1	0.1183 0.5363 0.3454

The expert group compares the risk factors of the rule layer in pairs and obtains the evaluation matrix X:


$$X_{5\times 5}=\left[\begin{array}{ccccc} \frac{x_{1}}{x_{1}} & \frac{x_{1}}{x_{2}} & \frac{x_{1}}{x_{3}} & \frac{x_{1}}{x_{4}} & \frac{x_{1}}{x_{5}}\\ \frac{x_{2}}{x_{1}} & \frac{x_{2}}{x_{2}} & \frac{x_{2}}{x_{3}} & \frac{x_{2}}{x_{4}} & \frac{x_{2}}{x_{5}}\\ \frac{x_{3}}{x_{1}} & \frac{x_{3}}{x_{2}} & \frac{x_{3}}{x_{3}} & \frac{x_{3}}{x_{4}} & \frac{x_{3}}{x_{5}}\\ \frac{x_{4}}{x_{1}} & \frac{x_{4}}{x_{2}} & \frac{x_{4}}{x_{3}} & \frac{x_{4}}{x_{4}} & \frac{x_{4}}{x_{5}}\\ \frac{x_{5}}{x_{1}} & \frac{x_{5}}{x_{2}} & \frac{x_{5}}{x_{3}} & \frac{x_{5}}{x_{4}} & \frac{x_{5}}{x_{5}} \end{array}\right]=\left[\begin{array}{ccccc} 1 & 1/2 & 1 & 1/3 & 1/3\\ 3 & 1 & 3 & 1 & 1/2\\ 1 & 1/3 & 1 & 1/2 & 1/3\\ 2 & 1 & 2 & 1 & 2\\ 3 & 1/2 & 3 & 1/2 & 1 \end{array}\right]$$

*W_i*(*i* = 1, 2, 3, 4, 5) is the weight vector of the factor layer (i.e., second-class index), which includes the origin warehouse risk, regional central warehouse risk indictors, front warehouse risk indictors, home delivery service risk indictors, and industry information platform risk indictors.*WX*_1_ = 0.1086; *WX*_2_ = 0.2616;*WX*_3_ = 0.1086;*WX*_4_ = 0.2935;*WX*_5_ = 0.2277.λ_*max*_ = 4.9418, $C\,I=\frac{\lambda_{\max}-n}{n-1}$ = 0.0145, $C\,R=\frac{C\,I}{R\,I}$ = 0.0129 <0.1, CR is obtained by calculation, which indicates that the judgment matrix of this evaluation has passed the consistency test, and the weight coefficient obtained by the AHP method is more reasonable.

**FIGURE 4:**
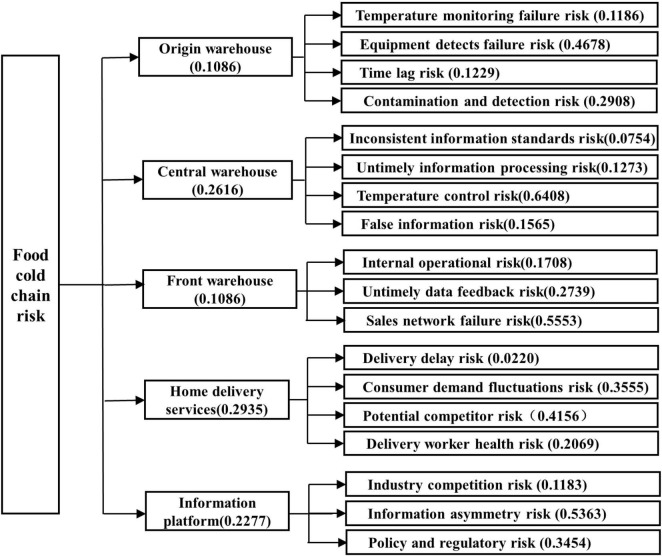
AHP weights of factors in layers 1–2.

Similarly, we can get the AHP weights of all indexes in the food cold chain risk index system by calculating layer by layer from top to bottom. The combined weights of the object layer, rule layer, and factor layer are shown in Figure 4.

By analyzing the common target factors, a pairwise comparison matrix is formed, and the weight set is obtained by calculating according to the above formula (2). CR <0.1 is obtained by calculation, which indicates that the judgment matrix of this evaluation has passed the consistency test, and the weight coefficient obtained by the AHP method is more reasonable.

### Analytic Hierarchy Process-Entropy Weight Method to Calculate the Weight of Each Index

When using the entropy weight method to calculate the weight of each index, the index information set of all samples are collected first and then the index weight of this evaluation sample is calculated according to formulas (8)–(11). In addition, the rule layer will be taken as an example to illustrate how to calculate. The calculation results are shown in Table 5.

**TABLE 5:** Entropy weight of the rule layer.

Rule layer	Origin warehouse	Regional central warehouse	Front warehouse	Home delivery services	Industry information platform
Entropy weight	0.1889	0.2116	0.1996	0.207	0.1929

Finally, the two weights are weighted and fused according to formula (12) to obtain the final combination weight of the rule layer index, as shown in Table 6.

**TABLE 6:** Comprehensive weights of the rule layer.

Rule layer	AHP weight $w_{i}^{{}^{\prime }}$	Entropy Weight ${\text{w}}_{i}^{{}^{\prime \prime }}$	Comprehensive weight
X_1_	0.1086	0.1889	0.1407
X_2_	0.2616	0.2116	0.2416
X_3_	0.1086	0.1996	0.1450
X_4_	0.2935	0.2070	0.2589
X_5_	0.2277	0.1929	0.2138

Similarly, the entropy weight of the evaluation matrix of each factor layer can be obtained. *W_i*(*i* = 1, 2, 3, 4, 5) is the weight vector of the factor layer (i.e., second-class index), which includes the origin warehouse risk, regional central warehouse risk indictors, front warehouse risk indexes, home delivery service risk indexes, and industry information platform risk indexes.

*W*_1_ = [0.1371, 0.2285, 0.1371, 0.8225]; *W*_2_ = [0.0998, 0.1597, 0.6389, 0.1996; *W*3 = [0.1663, 0.2772, 0.3104]; *W*_4_ = [0.0559, 0.3478, 0.5651, 0.1553]; *W*_5_ = [0.1504, 0.4061, 0.1354].

**FIGURE 5:**
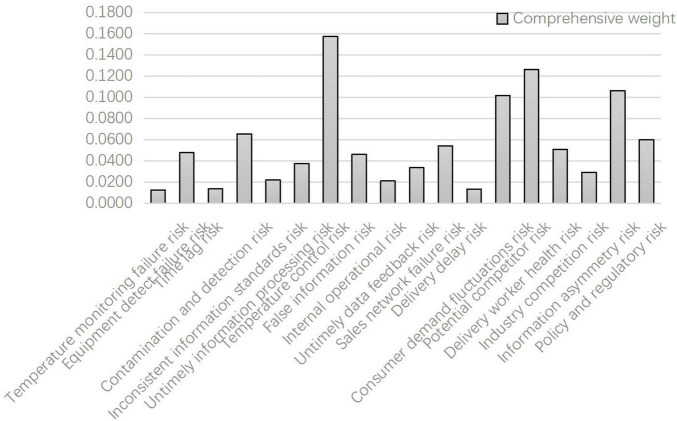
Comprehensive weights of each index by AHP-entropy weight method.

This article calculates the comprehensive weight coefficient; the coefficient α was selected as 0.6 according to the opinions of the expert group. The two weights were calculated and fused according to formula (12) to obtain the final combination weight of the rule layer index. The weight results of each index are shown in Table 7 and Figure 5.

**TABLE 7:** Weights of each index by AHP-entropy weight method.

Layer 1	Layer 2	AHP weights	Entropy weight	Comprehensive weight
Origin warehouse X_1_	Temperature monitoring failure risk	0.008	0.019	0.013
	Equipment detects failure risk	0.058	0.032	0.048
	Time lag risk	0.01	0.019	0.014
	Contamination and detection risk	0.032	0.116	0.066
Regional central warehouse	Inconsistent information standards risk	0.021	0.024	0.022
	Untimely information processing risk	0.037	0.039	0.037
	Temperature control risk	0.16	0.154	0.157
	False information risk	0.045	0.048	0.046
Front warehouse	Internal operational risk	0.019	0.024	0.021
	Untimely data feedback risk	0.029	0.04	0.034
	Sales network failure risk	0.06	0.045	0.054
Home delivery services	Delivery delay risk	0.013	0.014	0.013
	Consumer demand fluctuations risk	0.11	0.09	0.102
	Potential competitor risk	0.113	0.146	0.126
	Delivery worker health risk	0.058	0.04	0.051
Industry information platform	Industry competition risk	0.027	0.032	0.029
	Information asymmetry risk	0.12	0.087	0.106
	Policy and regulatory risk	0.081	0.029	0.06

To verify better the validity of the calculation results, *X*_23_ > *X*_43_ > *X*_42_ > *X*_52_ > *X*_44_ > *X*_53_ > *X*_33_ > *X*_14_ > *X*_12_ > *X*_24_ > *X*_22_ > *X*_32_ > *X*_21_ > *X*_31_ > *X*_51_ > *X*_11_ = *X*_13_ > *X*_41_. The evaluation results of the risk level obtained by the comprehensive weighting method based on the AHP-entropy weight method are very close to the expected results of the previous expert evaluation.

## Discussion

A safe and efficient cold chain system is jointly constructed by many professional and competitive enterprises. This article analyzes and evaluates the major risks faced by enterprises of China’s food cold chain in the non-epidemic situation and the COVID-19 situation.

### Discussion in the Non-epidemic Situation

In the non-epidemic situation, the major risks faced by enterprises of China’s food cold chain are temperature control risk, potential competitor risk, and consumer demand fluctuation risk.

(1) In terms of temperature control risk, different fresh foods have relatively strict requirements for the storage temperature and temperature fluctuation rate. Since there are many cold chain links, spanning a wide range of regions and lasting for a long time, and this temperature control is completely unnatural, it is necessary to apply the most modern advanced technology to achieve high-quality whole chain temperature control under efficient and excellent management. Therefore, the total investment of the food cold chain is very high, but it is necessary. If the technology equipment investment is insufficient, the organization of processes is not strict enough, and the quality monitoring is not in place, excessive temperature fluctuation will appear in any link, and the food will deteriorate. Some deterioration cannot be distinguished by the naked eye but will cause harm to the health of consumers, so temperature control is the highest risk.
(2) In terms of potential competitor risk of cold chain enterprises, consumers have great realistic and potential demand for cold chain services, which attracts a large number of potential competitors to enter the cold chain industry. From the characteristics of enterprises, the construction and operation costs of cold chain enterprises are very high because at least strict temperature control should be achieved throughout the whole chain. However, in the stage of market promotion and cultivation, it is unrealistic to increase the product price, and it is normal that losses are incurred in the initial stage.
  Before gaining enough market share, if competitors with strong financial strength continue to join the ranks of competition, the survival and development of cold chain enterprises are difficult. Therefore, the risk of potential competitors is great. Major e-commerce platforms with obvious advantages in big data and information networks, such as Alibaba, Jingdong (JD), Suning, and other well-known e-commerce platforms in China, are gradually establishing cold chain networks through self-construction or acquisition. In contrast, traditional express companies such as Shunfeng (SF Express), Zhongtong (ZTO Express), and Yuantong (YTO Express) are also actively developing cold chain networks based on express business. These companies bring great competitor risks to existing cold chain enterprises.
(3) In terms of consumer demand fluctuation risk, with the development of China’s economy, consumers’ purchasing power and consumption level are constantly improving, and consumers’ requirements for the variety, quantity, quality, and timeliness of cold chain services are increasing. Consumers’ demand will also change with the change of season and climate, with the fluctuation of the overall economic situation, as well as the huge difference in demand due to regional differences. Therefore, the construction and operation of cold chain enterprises need to face the risk of consumers’ demand fluctuation.

### Discussion in the Coronavirus Disease 2019 Situation

In the COVID-19 situation, the virus can survive at low temperatures and aggravate highly the risk of food cold chain. Once the product is polluted and lacks efficient and timely management and control, it will bring major safety accidents to the whole cold chain. Therefore, the following two risks become very important.

(1) Contamination and detection risk in the producing area is high. The products of food cold chain come from all over the world, and in the COVID-19 situation, the risk is great that products are contaminated at the source. Limited by the frequency and equipment of detection, the contaminated products are at risk of not being found in time. Once the contaminated products enter the middle and lower reaches of the food cold chain, it may trigger the serious systemic risk of the cold chain. For example, “imported cherry incident” and “overseas shrimp incident,” the products contaminated by viruses in the origin, were found in time during routine sampling inspection in the warehouse, and the products were destroyed in time. For a small number of products that have entered the cold chain, they are located and recalled in time through the modern information traceability system, so as to avoid serious food safety accidents.
(2) Delivery worker health risk is also high. In the case of COVID-19, in the distribution to the consumer link, the delivery workers contact directly a large number of consumers every day, and they also pass through many places, so the probability of infection is much higher than others. Once the delivery workers are infected with the virus, it may spread to many other people, resulting in a serious accident of the spread of the epidemic.

## Conclusion and Proposals

### Conclusion

In this article, the expert system, AHP, and entropy weight method were comprehensively applied to the risk assessment of food cold chain. Through the effective identification and assessment of high-risk factors of food cold chain, the major risks that have the greatest impact on the whole cold chain were found. Moreover, the structure and source of food cold chain risk were also well analyzed. The results are consistent with the actual situation of food cold chain. The following are the major risks faced by China’s food cold chain:

(1) In the non-epidemic situation, the major risks include temperature control risk, potential competitor risk, and consumer demand fluctuation risk.
(2) In the COVID-19 situation, “contamination and detection risk” and “delivery worker health risk” become particularly important.

### Proposals

Based on the above conclusions, the specific measures and proposals for the risk management and control of food cold chain are put forward in the situation of COVID-19 and non-epidemic, to solve the problem of cold chain quickly and effectively and further optimize the food cold chain and improve its value.

(1) In the non-epidemic situation, this article proposes that to ensure strict temperature control in the whole chain, cold chain enterprises should build a sufficient number of advanced front warehouses and high-technology professional transportation, and the end distribution link also needs to be equipped with miniaturized short-distance insulation equipment. At the same time, it is necessary to build a modern IoT information system covering the whole chain, implement the whole process real-time monitoring of temperature and humidity, including real-time alarm of temperature loss, and establish a traceability system. In view of the potential competitor risks, on the one hand, we should strive to improve the service quality, operation efficiency, and management level of enterprises. In contrast, we should establish enterprise alliances, integrate all high-quality resources, promote the formulation of industry norms, and construct a good industry environment. Facing the consumer demand fluctuation risk, we should apply a more flexible operation strategy.
(2) In the COVID-19 situation, this article proposes effective measures to reduce the risk of food cold chain.
  (a) Carrying out high-density sampling inspection in the source, storage, and distribution links to reduce the source contamination and detection risk. It is necessary to adopt advanced technology to develop and apply efficient batch quarantine equipment and portable quarantine equipment in time. Moreover, the technical training shall be carried out for quarantine personnel.
  (b) Strengthening the storage supervision of food cold chain. First, disinfecting the outer packaging of products to ensure safety and comprehensively disinfecting the storage environment to prevent contamination. Second, intensively disinfecting and supervising the transportation equipment and the clothing of front-line employees, and intensively disposing of the generated waste. Third, improving the storage capacity and reserving enough qualified commodities as much as possible, so as to fill the commodity vacancy at a critical juncture.
  (c) Ensuring the health of front-line employees and avoiding infection with COVID-19. These employees include terminal distribution personnel, warehouse staff, and source operators. Strengthening the regular physical examination and psychological guidance of employees, especially the continuous detection of body temperature or nucleic acid. Carrying out all kinds of contactless distribution in the distribution link.
  (d) Improving the information traceability mechanism and promoting information exchange and sharing. Tracking and recording the transportation and storage of bulk cold chain products by GPS positioning system. Using electronic labels, it is convenient for managers and consumers to query and trace the information of commodity quality, service quality, delivery time, and so on. Applying 5G high-speed wireless global communication network to monitor the operation details of each link of the food cold chain in real time.

## Data Availability Statement

The original contributions presented in the study are included in the article/supplementary material, further inquiries can be directed to the corresponding author/s.

## Ethics Statement

Ethical review and approval was not required for the study on human participants in accordance with the local legislation and institutional requirements. Written informed consent from the patients/participants OR patients/participants legal guardian/next of kin was not required to participate in this study in accordance with the national legislation and the institutional requirements.

## Author Contributions

Both authors listed have made a substantial, direct, and intellectual contribution to the work, and approved it for publication.

## Conflict of Interest

The authors declare that the research was conducted in the absence of any commercial or financial relationships that could be construed as a potential conflict of interest.

## Publisher’s Note

All claims expressed in this article are solely those of the authors and do not necessarily represent those of their affiliated organizations, or those of the publisher, the editors and the reviewers. Any product that may be evaluated in this article, or claim that may be made by its manufacturer, is not guaranteed or endorsed by the publisher.
